# The Development of Maternal Psychological Control in Early Adolescence: Maternal, Youth, and Neighborhood Antecedents

**DOI:** 10.1007/s10964-022-01642-0

**Published:** 2022-06-16

**Authors:** Duyen T. Trang, Tuppett M. Yates

**Affiliations:** Department of Psychology, University of California, Riverside, 900 University Ave., Riverside, CA 92521 USA

**Keywords:** Psychological control, Parent psychopathology, Youth externalizing problems, Neighborhood risks, Informant reports

## Abstract

Despite abundant research documenting negative associations between parental psychological control and youth adjustment, little is known about precursors of parental psychological control. The current study evaluated maternal, youth, and neighborhood predictors of changes in maternal psychological control across the transition to adolescence. Mother-youth dyads (*N* = 211, 50.2% female children; 46.4% Latinx, 17.5% Black, 11.4% white, and 24.7% multiracial) reported on maternal psychological control at youth ages 10 and 12. Controlling for youth ethnicity and race, family income-to-needs, and prior levels of maternal psychological control at age 10, structural equation models showed that maternal problems (i.e., anxiety, alcohol dependence, caregiving helplessness) predicted increases and youth externalizing problems (e.g., attention problems, rule-breaking) predicted decreases in maternal reports of psychological control. Neighborhood risks (i.e., poverty, crime, single-parent households) predicted increases in youth reports of maternal psychological control. Exploratory analyses by gender indicated that neighborhood risks predicted decreases in maternal reports of psychological control for girls, but increases in maternal reports of psychological control for boys. This study identified specific antecedents of maternal psychological control that can be targeted in future intervention efforts to reduce negative parenting to promote positive youth development.

## Introduction

Psychological control involves manipulative and interfering attempts to control youth’s socioemotional development through inhibiting and/or invalidating tactics, such as dismissing youth’s feelings or attacking their self-worth (Barber, 1996). An abundance of research has shown that these negative and restricting parenting practices hinder normative youth development by thwarting autonomy-seeking and identity formation processes (Luyckx et al., 2007; Soenens et al., 2010), which leads to maladaptive youth outcomes, such as internalizing and externalizing problems (see Scharf and Goldner, 2018 for review). Given the relatively greater malleability of newly formed adaptive organizations (Sroufe, 2009), identifying factors that contribute to the development of parental psychological control in early adolescence will inform interventions to mitigate these negative parenting effects on youth adjustment. Building on initial examinations of precursors of parental psychological control (i.e., Laird, 2011; Pettit et al., 2001; Soenens et al., 2006), this longitudinal study employed a sociodemographically diverse community sample of mother-youth dyads to evaluate the distinct contributions of maternal, youth, and neighborhood characteristics to the development of maternal psychological control across the transition to early adolescence using assessments when youth were 10 and 12 years of age. Further, given the potential for youth gender to influence the expression of parental psychological control (e.g., Bean and Northrup, 2009), the current study offered a novel evaluation of these antecedents in separate samples of girls and boys.

### Antecedents of Parental Psychological Control

Belsky’s (1984) *process model of parenting* holds that parental factors (e.g., psychological attributes), youth characteristics (e.g., effortful control), and sociocontextual features (e.g., neighborhood violence) influence parenting behaviors. To date, only two studies have evaluated Belsky’s (1984) framework as applied to expressions of parental psychological control over time. In the first study, examiner ratings of maternal harsh parenting at age 5 were related to youth reports of higher maternal psychological control at age 13, whereas maternal reports of harsh parenting and child externalizing problems at age 8 were each associated with higher maternal reports of psychological control 5 years later (Pettit et al., 2001). In the second study, youth attributes (i.e., depressed mood, rule-breaking, and pubertal development) predicted increases in youth reports of maternal psychological control, whereas maternal education, female gender, African American race, and single-parent family structure predicted increases in maternal reports of psychological control one year later (Laird, 2011). A recent review that applied Belsky’s (1984) framework to the investigation of parental psychological control identified several limitations of prior studies, including reliance on cross-sectional research designs, single informants (i.e., parent or youth reports), and/or single method assessments (i.e., self-reports; Scharf and Goldner, 2018). The current prospective investigation extended these prior studies and addressed ongoing research gaps by employing multiple informants (i.e., youth, mothers, and examiners) and multiple methods (i.e., self-reports, administrative census data) to evaluate latent maternal, youth, and neighborhood contributors to changes in youth and maternal reports of maternal psychological control from ages 10 to 12.

#### Parent factors

Several parent characteristics may contribute to the development of parental psychological control. For example, research suggests that parents’ psychological problems, including substance abuse, may impair parenting capacities and increase the use of negative parenting (e.g., Tildesley and Andrews, 2008), though specific relations to psychological control have not been evaluated until now. For instance, psychological difficulties may interfere with parents’ empathy and perspective-taking skills (Werner et al., 2016), which are essential to recognize and support youth autonomy. Consistent with this view, parents’ anxiety and maladaptive perfectionism were related to youth and parent combined reports of parental psychological control during middle school (Soenens et al., 2006). Moving to the level of parental beliefs about parenting, parents who feel helpless in the parent-child relationship may underestimate their competencies (Linde-Krieger and Yates, 2018), which, in turn, may prompt them to engage psychologically controlling tactics to gain a sense of power and control in the parent-youth relationship. Indeed, recent evidence suggests a strong, positive correlation between maternal reports of caregiving helplessness and a perceived lack of control over their children’s behavior (Grip, 2019). Finally, though prior research indicates that parental alcohol dependence is negatively related to the provision of parental support, discipline, and monitoring in adolescence (King and Chassin, 2004), relations between parental alcohol dependence and psychological control remain unclear. Following Belsky’s (1984) assertion that parents’ personality and psychological functioning are salient influences on parenting practices, the current study employed measures of maternal anxiety symptoms, alcohol dependence, and sense of caregiving helplessness to identify relations between *maternal problems* and the development of maternal psychological control in early adolescence.

#### Youth factors

Consistent with well-documented child effects (e.g., delinquent behaviors, irritability) on parenting practices (see Bell and Harper, 2020 for review), research suggests that various youth characteristics might influence the expression of parental psychological control. Although some research suggests that youth internalizing problems, such as anxiety and separation difficulties, may evoke parental psychological control (e.g., Albrecht et al., 2007), other evidence suggests that youth externalizing problems, such as oppositional and delinquent behaviors, may trigger psychologically controlling strategies as parents seek to manage youth’s negative behaviors (see Scharf and Goldner, 2018 for review). Prior research has documented both cross-sectional and longitudinal associations between delinquent behavior and later youth and maternal reports of psychological control (Barber, 1996). However, the absence of controls for prior levels of psychological control in these studies has limited causal conclusions regarding potential youth effects on the expression of parental psychological control. Indeed, the few investigations that have included prior measures of parental psychological control yielded inconsistent results. For example, some findings indicate that youth reports of physical aggression toward their peers predict increased levels of youth reports of parental psychological control two years later (Albrecht et al., 2007), but others do not support significant concurrent or prospective relations between youth’s rule-breaking behaviors and either youth or maternal reports of psychological control (Laird, 2011). To evaluate predictive relations from youth externalizing behaviors to maternal psychological control, this study drew on multiple informants (i.e., youth, mothers, examiners) to mitigate shared method variance (Stone et al., 2013) and held prior maternal psychological control at age 10 constant to evaluate prospective relations from youth externalizing problems at age 10 to changes in maternal psychological control from ages 10 to 12.

#### Neighborhood factors

Parenting processes are embedded in multiple and interacting systems of contextual influence (Luster and Okagaki, 2006), including neighborhood characteristics. That said, despite a robust literature connecting neighborhood quality to parental *behavioral* control (e.g., rule enforcement and limit setting; Cuellar et al., 2015), surprisingly little research has studied the influence of neighborhood characteristics on the development of parental psychological control. Just as unpredictable and unsafe neighborhoods contribute to more restrictive behavioral practices to keep youth safe (e.g., increased parental monitoring; Leventhal & Brooks-Gunn, 2004), so, too, may they encourage parental psychological control. The *family stress model* (Conger et al., 1994) purports that unpredictable, unsafe, or low-resource contexts may hamper parents’ physical, mental, and material capacities to support positive development. Thus, neighborhood violence or economic disadvantage may result in elevated parental psychological control as parents strive to manage youth’s behavior and ensure their safety. This study evaluated the specific contribution of *neighborhood risks* as indicated by the amount of crime, poverty, and single-parent households to the development maternal psychological control.

### Antecedents of Parental Psychological Control by Youth Gender

Prior research has shown inconsistent gender patterns in rates of parental psychological control with some evidence showing elevated rates toward boys (e.g., Fu and Zhang, 2020), others toward girls (e.g., Smetana and Daddis, 2002), and still others showing no significant differences in parental psychological control rates by youth gender (e.g., Cui et al., 2014). Mirroring the dearth of research on antecedents of parental psychological control generally, few studies have evaluated youth gender differences in predictors of parental psychological control. Regarding maternal characteristics, girls with mothers with low levels of empathic concern and perspective-taking reported higher rates of maternal psychological control across adolescence (ages 13–18), but this relation was significant for boys only in late adolescence (ages 15–18; Werner et al., 2016). Similarly, relations between maternal reports of maladaptive perfectionism and youth reports of maternal psychological control did not significantly vary as a function of youth gender (Laird, 2011). Studies have not yet examined gender differences in predictions from youth externalizing problems or neighborhood characteristics to parental psychological control, but broader research on youth and neighborhood effects suggests they may differ by gender. For example, some data suggest that girls’ externalizing behaviors are negatively related youth reports of parental support and control (Huh et al., 2006), whereas other findings indicate that boys’, but not girls’, delinquent behaviors and school problems are negatively related to authoritative parenting (Kerr et al., 2012). Given the potential for differential influences of maternal, youth, and neighborhood factors on maternal psychological control directed toward girls versus boys, and in light of scarce research examining gender differences to date, this investigation explored the antecedents of maternal psychological control by youth gender.

## Current Study

The current investigation of theoretically-specified antecedents of maternal psychological control as suggested by Belsky’s (1984) *process model of parenting* fills prominent gaps in current research. First, the use of a longitudinal research design to evaluate antecedents of maternal psychological control from ages 10 to 12 offered more support for causal conclusions than previous cross-sectional studies. Second, using a structural equation modeling approach, multiple measures indicated all proposed antecedents (i.e., maternal problems, youth externalizing problems, and neighborhood risks) to provide the most comprehensive, yet parsimonious, assessment of each construct (e.g., maternal problems were indicated by maternal anxiety, alcohol dependence, and feelings of helplessness in the mother-youth relationship). Although structural equation modeling does not show the specific contribution of each indicator to maternal psychological control, this approach is preferable to traditional regression techniques because it explicitly includes measurement error in the model and evaluates the fit of the data against the proposed theoretical model (Kaplan, 2008). Third, multiple informants contributed to antecedent measures when possible (e.g., youth externalizing problems were indicated by youth, maternal, and examiner reports) to mitigate shared method variance concerns. Likewise, the proposed process model of maternal psychological control was evaluated with respect to both youth and maternal reports of psychological control. Fourth, the current study offered the first test of neighborhood risks as an antecedent of maternal psychological control. In sum, this longitudinal, multi-method, and multi-informant investigation tested a novel structural equation model to evaluate the overarching hypothesis that maternal problems, youth externalizing problems, and neighborhood risks would predict increases in youth and maternal reports of maternal psychological control from ages 10 to 12. Further, exploratory analyses evaluated the proposed model separately for girls and boys.

## Method

### Participants

Participating families (*N* = 211; 50.2% female youth) were drawn from an ongoing longitudinal study of child development. The sample was diverse with respect to ethnicity and race with 46.4% of youth identified as Latinx, 17.5% as Black, 11.4% as white, and 24.7% as multiracial. Participants were similarly diverse with regards to economic status with 36.5% of the families qualifying for government subsidies (e.g., food stamps) at age 10. The ethnic, racial, and economic composition of the sample represented the southern California region from which the sample was recruited (U.S. Census Bureau, 2014a).

Participating mothers were largely biological mothers (88.2%), foster/adoptive mothers (3.3%), and other kin (e.g., grandmothers; 8.5%) serving in the maternal role. At the time of the age 10 assessment, most mothers were married or in a committed relationship (75.3%), had completed at least a high school degree (79.7%), and were employed (58.2%). Dyads were selected for the current analyses if they completed laboratory assessments when youth were age 10 (*n* = 204, *M*_age_ = 9.61 years; *SD* = 0.27) and/or 12 (*n* = 197, *M*_age_ = 12.24, *SD* = 0.35).

### Procedures

Families were recruited through community-based childcare programs via flyers inviting participation in a longitudinal study of early learning and development. Mothers completed a brief phone intake screening and were excluded if the child had received a diagnosis of a developmental disability or delay (*n* = 3), was not able to complete the assessment in English (*n* = 4), and/or was not within the target age range at the start of the study (i.e., 45–54 months, not tracked). Data for the current analyses were collected from 2013 to 2018 across two data waves. At each wave, dyads completed a 3-h laboratory assessment, which consisted of various youth and parent surveys and observational tasks to assess youth’s representations of themselves, their mothers, and the mother-youth relationship. Each dyad member (i.e., youth and mother) was verbally interviewed by an individual examiner to reduce concerns about reading comprehension. Examiners were doctoral students and post-baccalaureate staff members who received training and ongoing supervision from the last author. Mothers were compensated with $25/h of assessment, and youth received a gift after each visit. Informed consent and assent were obtained from the mother and the youth, respectively. All procedures received approval from the human research review board of the participating university.

### Measures

#### Maternal psychological control

At ages 10 and 12, youth and mothers reported on maternal psychological control using Barber’s (1996) 8-item Psychological Control Scale (PCS). The PCS assesses parental attempts to restrict youth’s verbal expression, dismiss or manipulate youth’s feelings, pressure the youth to agree with the parent’s agenda, attack or embarrass the youth based on their behaviors, and induce guilt (e.g., “My parent acts like she knows what I’m thinking or feeling,” “My parent brings up past mistakes when she criticizes me”). Youth and mothers rated the PCS items on a 3-point Likert scale from *not like me/her* (1), *somewhat like me/her* (2), and *a lot like me/her* (3). Items were summed to compute a total index of psychological control, with higher scores connoting greater maternal psychological control. The PCS evidenced acceptable to good reliabilities at ages 10 (*α*_mother_ = 0.619; *α*_youth_ = 0.717) and 12 (*α*_parent_ = 0.702; *α*_youth_ = 0.641), and these were comparable to prior studies with diverse parent and youth samples (e.g., Nelson and Crick, 2002; Pettit et al., 2001; Tholia and Suri, 2020).

#### Maternal problems

At age 10, mothers reported their anxiety symptoms and alcohol dependence on the Millon Clinical Multiaxial Inventory (MCMI-III; Millon, 1997). Symptoms of anxiety were reported as *true* (1) or *false* (0) across 14 items (e.g., “Lately, I’ve been sweating a great deal and feel very tense;” *α* = 0.807). Symptoms of alcohol dependence were indicated by 15 items (e.g., “I have an alcohol problem that has made difficulties for me and my family;” *α* = 0.611). Upon further inspection, removing one item focused on work difficulties (i.e., “Drinking alcohol has never caused me any real problems in my work”) substantially improved the reliability of this subscale (*α* = 0.767), and made sense given 41.7% of the mothers in this study did not work outside the home. Items on each subscale were summed to indicate the frequency of anxiety symptoms and alcohol dependence, with higher scores suggesting greater anxiety symptoms and alcohol dependence.

Mothers reported their feelings of helplessness when parenting using the 6-item helpless subscale of the Caregiving Helplessness Questionnaire (CHQ; *α* = 0.681; George and Solomon, 2007). The CHQ taps the extent to which mothers feel helpless in the mother-youth relationship (e.g., “When I am with my child, I often feel out of control”) on a 5-point Likert scale from *not at all characteristic* (1) to *very characteristic* (5). Responses were composited across all items with higher scores denoting mothers who felt more helpless. Prior research supports the validity of this scale for use with early adolescent samples (Lecompte and Moss, 2014).

#### Youth externalizing problems

At age 10, youth and mothers reported youth externalizing and disruptive behaviors on the Diagnostic Interview Schedule for Children – IV (DISC-IV; Shaffer et al., 2000). The DISC is a structured diagnostic instrument focused on childhood psychiatric disorders with well-established reliability and validity. Youth and mothers completed parallel items to indicate the presence of inattention/hyperactivity (i.e., a composite of 23 items; “During this school year, did you/[youth] often dislike doing things where you/they had to pay attention for a long time?”, “Do you/Does [youth] often have trouble staying in your/their seat at school?”) and oppositional defiant behavior (i.e., a composite of 12 items; “In the last year, did you/[youth] often lose your/their temper?”). Youth and maternal reports of youth externalizing problems were indicated by the total number of endorsed symptoms.

Examiners reported on youth externalizing problems using the Test Observation Form (TOF; McConaughy and Achenbach, 2004). Following the 3-hour in-person assessment at age 10, examiners indicated the presence of youth externalizing behaviors (i.e., “temper tantrums, hot temper, or seems very angry,” “defiant, talks back, or sarcastic”) on a scale from *no occurrence* (0) to *definite, severe, high frequency occurrence* (3) across 34 items (*α* = 0.917). Items were summed, with higher scores indicating greater youth externalizing behaviors. The TOF is a psychometrically sound measure that has been widely used in sociodemographically diverse community and clinical samples (McConaughy and Achenbach, 2004).

#### Neighborhood risks

Three neighborhood risk indicators were obtained using the home address of the family at age 10. First, Uniform Crime Reports (UCR), which are reported annually to the Federal Bureau of Investigation (Federal Bureau of Investigation FBI (2014)), provided information about incidents of violent crime in the child’s city. Crime risk was measured as the total number of crime incidents in the child’s city divided by the total number of crime incidents in the state such that a score of 1 denoted average risk, while those above 1 indicated the degree to which the child’s city exceeded state-level crime rates. Poverty risk was calculated as the percentage of poverty in the family’s zip code divided by the percentage of poverty in the state using data from the American Community Survey (ACS; U.S. Census Bureau, 2014b). Single-parent household risk was obtained using the decennial census data (U.S. Census Bureau, 2010) and calculated as the percentage of single-parent households in the child’s zip code divided by the percentage of single-parent homes in the state. For each risk indicator, higher values denoted greater risk. Administrative records of neighborhood features based on cities and zip codes have been used widely in prior studies of neighborhood effects (see Leventhal and Dupéré, 2019 for review).

#### Family income-to-needs

At age 10, mothers reported their household income and any other income sources (e.g., child support) over the past year. Family income was divided by the poverty threshold in accordance with the household size and number of children under 18 years old who lived in the home to calculate the family income-to-needs (U.S. Census Bureau, 2014b) with higher values characterizing greater levels of family income-to-needs.

### Analytic Plan

Descriptive statistics and bivariate relations were computed in SPSS Version 27. Structural equation modeling analyses were conducted using the lavaan package in RStudio (Rosseel, 2012). Data were missing for 7.6% of all data points across variables at both data waves. Both youth and maternal reports of psychological control were missing at ages 10 (*n*_*youth*_ = 9, 4.3%; *n*_*mother*_ = 12, 5.7%) and 12 (*n*_*youth*_ = 24, 11.4%; *n*_*mother*_ = 22, 10.4%) because the participants did not complete the assessment. At age 10, data on youth characteristics were missing for externalizing problems as reported by youth (*n* = 17, 8.1%), mothers (*n* = 11, 5.2%), and examiners (*n* = 12, 5.7%). Data on parent characteristics at age 10 were missing for anxiety symptoms (*n* = 16, 7.6%), alcohol dependence problems (*n* = 16, 7.6%), and feelings of helplessness (*n* = 10, 5%). Neighborhood risk data from age 10 were missing for crime reports (*n* = 15, 7.1%), poverty rates (*n* = 49, 23.2%), and single-parent household structure (*n* = 21, 9.9%). Finally, eight families were missing information on income-to-needs because they did not complete the assessment (*n* = 7, 3.3%) or provided insufficient data (*n* = 1; 0.01%). Full information maximum likelihood (FIML) estimation in RStudio accounted for missing data as supported by Little’s (1988) missing completely at random (MCAR) test, *χ*^*2*^ (460) = 484.20, *p* = 0.21. Further, the robust Maximum Likelihood estimator in lavaan addressed non-normality in manifest variables.

The first set of structural equation modeling analyses specified the measurement model using a chi-square difference test to evaluate a one-factor confirmatory factor analysis (CFA) with all manifest variables as compared to the hypothesized three-factor CFA in which each latent factor was formed by the proposed manifest variables (i.e., maternal anxiety symptoms, alcohol dependence symptoms, and feelings of helplessness indicated maternal problems; youth, maternal, and examiner reports of youth externalizing problems indicated youth externalizing problems; and neighborhood crime risk, poverty risk, and single-parent household risk indicated neighborhood risks). Good model fit was determined by root mean square error of approximation (RMSEA) and standardized root mean square residual (SRMR) values below 0.08, and comparative fit index (CFI) and Tucker Lewis Index (TLI) values above 0.90 (Hu and Bentler, 1999). The second set of structural equation modeling analyses evaluated the unique contributions of parent problems, youth externalizing problems, and neighborhood risks at age 10 to changes in youth and maternal reports of psychological control from ages 10 to 12. Follow-up multigroup analyses explored the proposed model separately for girls and boys. When there was a significant gender difference, the fit of a constrained model wherein the path of interest was fixed to be equal between girls and boys was compared to the fit of an unconstrained model wherein all parameters were freely estimated. A significant chi-square difference test in the fit of constrained versus unconstrained models indicated that the path varied significantly between girls and boys. All analyses controlled for prior reports of maternal psychological control at age 10, family income-to-needs, and youth ethnicity and race.

## Results

### Descriptive Statistics and Bivariate Relations

Table 1 presents descriptive statistics and bivariate relations among study variables. Overall, maternal problems (i.e., anxiety symptoms, alcohol dependence, and feelings of helplessness) were positively associated with one another, as well as with maternal and examiner reports of youth externalizing problems, and with youth and maternal reports of psychological control at ages 10 and 12. Regarding youth externalizing problems, examiner reports of externalizing problems were positively related to both youth and maternal reports of externalizing, but youth and maternal reports of externalizing problems did not significantly correlate with each other. All informant reports of youth externalizing problems positively correlated with youth reports of maternal psychological control at age 10. Maternal reports of externalizing problems were positively associated with maternal reports of psychological control at ages 10 and 12, whereas youth and examiner reports of externalizing problems were positively related only to youth reports of maternal psychological control at age 12. Neighborhood risks (i.e., crime risk, poverty risk, and single-parent household risk) were positively correlated with one another. Crime risk was positively related to youth reports of maternal psychological control, but negatively associated with maternal reports of psychological control at age 12. Youth and maternal reports of psychological control were positively correlated within and across time, with one exception, which was that maternal reports of psychological control at age 10 did not significantly correlate with youth reports of maternal psychological control at age 12. Finally, family income-to-needs was negatively related to maternal alcohol dependence, crime risk, and single-parent household risk, but positively related to youth reports of externalizing problems.

**Table 1:** Descriptive statistics and bivariate correlations among study variables (*N* = 211)

Variable	*M* (*SD*)	﻿1	﻿2	3	4	5	6	7	8	9﻿	10	11	12	13
*Age 10*														
1. M Anxiety symptoms	0.17 (0.25)	−												
2. M Alcohol dependence	0.09 (0.15)	0.614***	−											
3. M Feelings of helplessness	7.25 (2.25)	0.501***	0.450***	−										
4. Y Youth reports of externalizing problems	2.04 (1.86)	−0.015	0.043	0.078	﻿−									
5. Y Maternal reports of youth externalizing problems	3.17 (2.35)	0.280***	0.275***	0.282***	0.101	−								
6. Y Examiner reports of youth externalizing problems	8.06 (10.33)	0.220**	0.203**	0.254***	0.205**	0.446***	﻿−							
7. N Crime risk	1.10 (0.23)	−0.088	−0.026	−0.054	−0.055	0.005	0.105	﻿−						
8. N Poverty risk	1.27 (0.36)	0.038	0.102	0.026	−0.038	0.085	−0.005	0.273**	−					
9. N Single-parent risk	1.33 (0.34)	−0.024	0.013	−0.003	−0.095	0.116	0.028	0.233**	0.315***	−				
10. Family income-to-needs	1.91 (1.35)	−0.105	−0.096	−0.083	0.172*	−0.042	0.016	−0.085	−0.194*	−0.209*	﻿−			
11. Youth reports of maternal psychological control	1.40 (0.37)	0.076	0.219**	0.237**	0.268***	0.197**	0.234**	−0.027	−0.110	−0.033	−0.017	−		
12. Maternal reports of maternal psychological control	1.22 (0.24)	0.385***	0.288***	0.386***	−0.004	0.247***	0.059	−0.081	−0.111	0.043	−0.093	0.141*	−	
*Age 12*														
13. Youth reports of maternal psychological control	1.39 (0.32)	0.051	0.204**	0.202**	0.155*	0.070	0.156*	0.156*	0.048	0.113	−0.003	0.405***	0.075	−
14. Maternal reports of maternal psychological control	1.25 (0.27)	0.324***	0.360***	0.514***	0.111	0.158*	−0.051	−0.194*	0.021	−0.031	0.027	0.212**	0.574***	0.190*

*M* maternal problems indicator, *Y* youth externalizing problems indicator, *N* neighborhood risk indicator

*p < 0.05, **p < 0.01, ***p < 0.001

### Measurement model

**Fig. 1:**
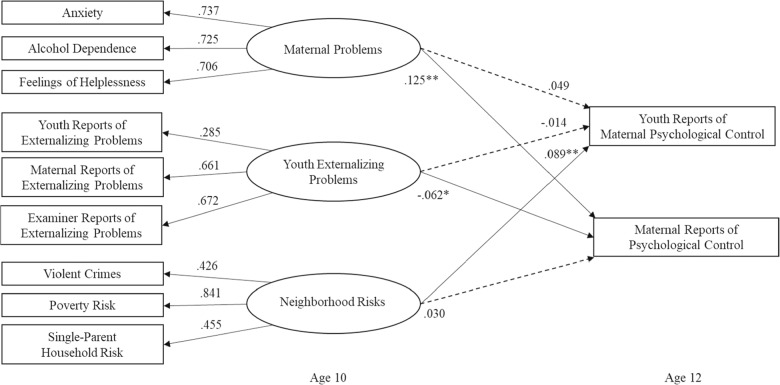
Factor loadings and regression coefficients for the total sample. *Note*. Standardized coefficients are presented in the model. Solid lines convey significant relations, whereas dashed lines indicate nonsignificant relations. *p < 0.05. **p < 0.01

A one-factor CFA model with all nine manifest variables was evaluated against a three-factor CFA model with each factor indicated by three manifest variables. The three-factor CFA model of maternal problems, youth externalizing problems, and neighborhood risks fit the data well, *χ*^*2*^ (24) = 21.13, *p* = 0.63, RMSEA < 0.001, SRMR = 0.04, CFI = 1.00, TLI = 1.02, and significantly better than the one-factor CFA model, *χ*^*2*^ (27) = 91.75, *p* < 0.001, RMSEA = 0.11, SRMR = 0.10, CFI = 0.75, TLI = 0.67, as confirmed by a significant chi-square difference test, Δ*χ*^*2*^ (3) = 70.63, *p* < 0.001. All factor loadings in the three-factor CFA model were significant (see Fig. 1). Although the factor loading of 0.285 for youth reports of externalizing problems was relatively low, we retained this indicator in the final model because of its theoretical relevance (e.g., Afthanorhan, 2014), and as supported by follow-up sensitivity analyses.

### Structural Equation Modelling for the Total Sample

Structural equation model analyses indicated good model fit when evaluating the unique contributions of maternal problems, youth externalizing problems, and neighborhood risks to changes in youth and maternal reports of psychological control from ages 10 to 12 while accounting for prior levels of maternal psychological control at age 10, family income-to-needs, and youth ethnicity and race, *χ*^*2*^ (63) = 115.56, *p* < 0.001, RMSEA = 0.06, SRMR = 0.06, CFI = 0.90, TLI = 0.83. As shown in Table 2 and Fig. 1, maternal problems predicted significant increases in maternal reports of psychological control at age 12 in the total sample, over and above prior maternal reports of psychological control at age 10. However, this path was not significant for youth reports of maternal psychological control. Youth externalizing problems predicted declines in maternal reports of psychological control from ages 10 to 12, but no significant changes in youth reports of maternal psychological control. Neighborhood risks predicted significant increases in youth reports of maternal psychological control, but did not predict significant changes in maternal reports of psychological control.

**Table 2:** Maternal problems, youth externalizing problems, and neighborhood risks at age 10 as predictors of changes in youth and maternal reports of maternal psychological control from ages 10 to 12 for the Total Sample (*N* = 211), Girls (*N* = 106), and Boys (*N* = 105)

	Youth reports of maternal psychological control (age 12)				Maternal reports of maternal psychological control (age 12)			
	*b*	*B*	*SE*	*p*	*b*	*B*	*SE*	*p*
Maternal problems	**0.049**	**0.152**	**0.033**	**0.134**	**0.125**	**0.467**	**0.028**	**<0.001**
	−0.*042*	−0.*132*	*0.050*	*0.393*	*0.024*	*0.102*	*0.036*	*0.509*
	0.090	0.276	0.043	0.037	0.133	0.450	0.038	0.001
Youth externalizing problems	**−0.014**	**−0.043**	**0.038**	**0.718**	**−0.062**	**−0.231**	**0.027**	**0.021**
	*0.060*	*0.186*	*0.072*	*0.404*	*0.018*	*0.077*	*0.020*	*0.544*
	−0.003	−0.008	0.051	0.958	−0.087	−0.295	0.048	0.072
Neighborhood risks	**0.089**	**0.276**	**0.031**	**0.005**	**0.030**	**0.113**	**0.023**	**0.180**
	*0.143*	*0.445*	*0.045*	*0.001*	−0.*061*	−0.*2﻿60*	*0.027*	*0.024*
	0.056	0.171	0.046	0.226	0.097	0.327	0.048	0.044
Youth reports of maternal psychological control (age 10)	**0.129**	**0.397**	**0.024**	**<0.001**				
	*0.077*	*0.251*	*0.047*	*0.102*				
	0.139	0.403	0.032	<0.001				
Maternal reports of maternal psychological control (age 10)					**0.114**	**0.426**	**0.019**	**<0.001**
					*0.135*	*0.507*	*0.029*	<*0.001*
					0.143	0.530	0.028	<0.001
Family income-to-needs	**0.052**	**0.160**	**0.027**	**0.052**	**0.051**	**0.192**	**0.019**	**0.008**
	*0.027*	*0.078*	*0.039*	*0.480*	*0.032*	*0.126*	*0.024*	*0.179*
	0.053	0.173	0.035	0.127	0.080	0.290	0.034	0.019
Youth ethnicity-race	**0.023**	**0.036**	**0.045**	**0.604**	**0.007**	**0.014**	**0.033**	**0.822**
	*0.029*	*0.045*	0.*065*	0.651	−0.*05﻿0*	−0.*105*	*0.041*	*0.224*
	0.062	0.094	0.061	0.308	0.011	0.019	0.053	0.836
Model *R*^2^	**0.243**, ***p*** < **0.001, ƒ**^**2**^ = **0.321**				**0.472**, ***p*** < **0.001, ƒ**^**2**^ = **0.894**			
	*0.356, p* < *0.001, ƒ*^*2*^ = *0.553*				*0.447, p* < *0.001, ƒ*^*2*^ = *0.808*			
	0.338, *p* < 0.001, ƒ^2^ = 0.512				0.542, *p* < 0.001, ƒ^2^ = 1.183			

Ethnicity-race was coded as 0 (non-Latinx) and 1 (Latinx)

Bold font = total sample; italicized font = girls; normal font = boys

### Structural Equation Modelling by Youth Gender

Exploratory multigroup analyses by youth gender revealed that the unconstrained model with all paths freely estimated demonstrated good model fit, *χ*^*2*^ (126) = 164.30, *p* = 0.012, RMSEA = 0.05, SRMR = 0.06, CFI = 0.92, TLI = 0.87. Maternal problems predicted significant increases in both youth and maternal reports of psychological control among boys, but not among girls. Further, chi-square difference tests evaluating unconstrained versus constrained models were marginally significant for both youth and maternal reports, *Δχ*^*2*^ (1) = 3.73, *p* = 0.053, *Δχ*^*2*^ (1) = 3.55, *p* = 0.060, respectively, which indicates that these paths differed marginally by youth gender. In addition, although not significant in the total sample, neighborhood risks predicted increases in girls’, but not boys’, reports of maternal psychological control. However, the chi-square difference test comparing the unconstrained model to the constrained model was not significant, *Δχ*^*2*^ (1) = 1.52, *p* = 0.218, which indicates that this path did not vary significantly between girls and boys. Neighborhood risks also predicted significant decreases in maternal reports of psychological control toward girls, but increases in maternal reports of psychological control toward boys. A significant chi-square difference test comparing the unconstrained and constrained models, *Δχ*^*2*^ (1) = 8.03, *p* = 0.005, indicates that this path varied meaningfully by youth gender.

### Sensitivity Analyses

Follow-up sensitivity analyses were performed in the total sample and multigroup tests to consider the removal of youth reports of externalizing problems considering its relatively low loading in the models. All findings replicated fully when only maternal and examiner reports were used to indicate youth externalizing problems. Hence, due to its theoretical relevance, youth reports of externalizing problems were retained in the final models.

## Discussion

Despite numerous studies demonstrating negative developmental effects of parental psychological control in adolescence (see Scharf & Golder, 2018 for review), less is known about precursors contributing to parental psychological control. Following Belsky’s (1984) *process model of parenting*, the current study documented specific contributions of maternal problems, youth externalizing problems, and neighborhood risks to changes in youth and maternal reports of maternal psychological control across the transition to early adolescence (i.e., ages 10 to 12), while controlling for prior reports of maternal psychological control at age 10, family income-to-needs, and youth ethnicity and race. Study results revealed distinct antecedents of maternal psychological control depending on the identity of the informant. Maternal problems and youth externalizing problems predicted increased and decreased maternal reports of psychological control, respectively. Neighborhood risks predicted increased youth reports of maternal psychological control. Exploratory analyses by youth gender indicated that maternal problems predicted increased youth and maternal reports of psychological control for boys only, whereas neighborhood risks predicted decreased maternal reports of psychological control for girls, but increased youth reports of maternal psychological control for boys.

As hypothesized, maternal problems emerged as a significant precursor to increased maternal reports of psychological control across the transition to early adolescence in this sample. This pattern corroborates prior suggestions that parents’ psychological functioning may be the most powerful antecedent of psychological control (Barber and Harmon (2002), and is consistent with previous research showing that parents who experience mental health difficulties may struggle to support their youth’s age-appropriate bids for autonomy during early adolescence (Werner et al., 2016). Maternal anxiety, alcohol dependence, and/or feelings of helplessness in the parent-youth relationship may interfere with mothers’ capacities not only to respond sensitively to youth’s cues for independence, but also to navigate the stress associated with normative changes in mother-youth dynamics leading to increased use of maternal psychological control to manage their youth and maintain a familiar mother-youth relational dynamic.

In contrast to study hypotheses, youth externalizing problems predicted decreased maternal reports of psychological control across the transition to early adolescence. This pattern contradicts some data indicating that youth externalizing problems may be positively related to youth reports of parental psychological control (Albrecht et al., 2007), though this study examined older youth (ages 12–19) and relied on youth reports of both aggression and parental psychological control. Despite mixed findings, the current study speaks to the ongoing need to consider potential youth effects in efforts to understand parental psychological control (and other parenting practices) across development (e.g., Reitz et al., 2006). Although this negative relation awaits replication, it may be that mothers redirect their control tactics toward more behavioral strategies as youth’s externalizing behaviors increase. Future studies that consider both psychological *and* behavioral control practices will help to clarify these relations further.

The current study joins prior research showing robust relations between perceived neighborhood quality and reports of parental *behavioral* control (e.g., Deutsch et al., 2012), to demonstrate that objective neighborhood risks also influence expressions of maternal psychological control in early adolescence. Youth reported higher rates of maternal psychological control as their neighborhood risk levels increased. These findings are consistent with the *family stress model* (Conger et al., 1994), which posits that parents living in impoverished and unsafe neighborhoods may experience stressors that prompt psychological control practices to protect youth from harm, and these practices may increase as youth engage in more activities outside the home across early adolescence. Although this investigation did not include assessments of behavioral control, as noted earlier, it will be interesting to see if and how these relations vary across different expressions of parental control over time.

When the proposed antecedents of maternal psychological control were examined across youth gender groups, maternal problems were significantly related to youth and maternal reports of psychological control among boys, but neither pathway was significant for girls. The obtained findings may reflect the reliance on female caregivers in the current study, particularly given some evidence suggesting that boys may be more sensitive to maternal mental health struggles than girls (Biederman et al., 2002). In addition, as adolescence dawns, mothers struggling with psychological difficulties may feel particularly threatened by their sons’ increasing capacity to function with more autonomy prompting them to engage psychological control tactics to keep their sons emotionally close to them and retain control over the mother-son relationship. For example, mothers’ dependency was positively related to controlling behaviors (i.e., explicit commands during an interaction task) when they perceived their adolescent sons as less competent in their problem-solving skills (Thompson and Zuroff, 1998). Although these findings suggest that psychologically vulnerable mothers may increase their control tactics when they feel they are at risk of losing their connection to their sons, the marginal significance of model comparisons by gender in this study points to the need for further replication in future research.

Neighborhood risks predicted decreased maternal reports of psychological control toward girls, but increased maternal reports of psychological control toward boys. One explanation for this pattern is that mothers may employ behavioral control to keep girls close to home in the context of neighborhood risk, whereas they may engage in higher psychological control toward boys precisely because they feel less able to control boys’ behaviors, especially given that boys venture out into the community more frequently than girls (Clampet-Lundquist et al., 2011).

### Limitations and Future Directions

The investigation evaluated prospective relations from latent maternal, youth, and neighborhood factors to changes in both youth and maternal reports of psychological control from childhood to early adolescence using a robust structural equation modelling analytic approach. Moreover, the use of multiple informants (i.e., youth, parent, examiner, and administrative data) advanced beyond single informant limitations to address shared method variance concerns that characterize many prior studies of parental psychological control. Finally, exploratory analyses by youth gender highlighted additional complexities that warrant consideration in future research on parental psychological control. Despite these contributions, several limitations qualify the study findings while illuminating promising directions for future research.

First, although the inclusion of prior controls for maternal psychological control when youth were 10 years old provided some support for causal interpretations of the current findings, additional data waves with multiple measures at all time points are needed to evaluate the likely bidirectional relations between maternal psychological control and antecedent factors. For instance, prior research supports relations from parental psychological control to youth maladjustment (see Scharf and Goldner, 2018 for review), as well as from youth adjustment problems to parental psychological control (Pettit et al., 2001). Using cross-lagged analyses, some researchers have found reciprocal relations between parental psychological control and youth behavior problems (He et al., 2019), whereas others have not (Gao et al., 2021). Bidirectional relations may also be relevant for understanding associations between parent problems and psychological control practices. However, such bidirectionality in relations with neighborhood risks is less likely, since parenting practices would not be expected to influence the neighborhood risks examined here.

Second, although the PCS (Barber, 1996) is the most widely used measure of parental psychological control in diverse samples, the PCS evidenced modestly reliable youth reports at age 12 and parent reports at age 10 in the current sample. Of note, the obtained reliabilities in this study are consistent with prior research using parent reports on the PCS, which published alpha reliabilities of 0.63 (Pettit et al., 2001), 0.64 (Tholia and Suri, 2020), and 0.65 (Nelson and Crick, 2002). In addition to raising concerns that the current findings may underestimate the actual magnitude of relations between identified antecedent factors and changes in maternal psychological control, this and prior studies demonstrate the pressing need for ongoing efforts to develop and validate reliable measures of parental psychological control.

Third, as discussed earlier, the current focus on female caregivers likely influenced the obtained findings, particularly gender-specific relations, in ways that could not be evaluated fully. Amidst growing recognition that parent and youth gender influence both antecedents and outcomes of parenting processes (Tasker, 2010), it is critical to expand ongoing research on psychological control to consider both fathers and mothers, as well as sons and daughters. For instance, Lansford et al. (2014) found that when youth reports of maternal and paternal psychological control were included in the same model, only paternal psychological control predicted increases in youth’s internalizing and externalizing problems.

Fourth, maternal, youth, and neighborhood characteristics likely operate in complex ways to influence psychological control. For instance, a recent review of research guided by Belsky’s (1984)’s *process model of parenting* emphasized the importance of contextual stressors (i.e., financial problems, low social support, ethnic-racial discrimination) in magnifying relations between parent problems and negative parenting practices (Taraban and Shaw, 2018). Similarly, in line with a prior review noting a negative association between adverse neighborhood contexts (i.e., neighborhood danger, disadvantage, and disengagement) and positive parenting (Cuellar et al., 2015), neighborhood risks may contribute to parent mental health problems (e.g., depression, anxiety; Self-Brown et al., 2006), which, in turn, may affect parental psychological control in ways that could not be examined here. Future research on parental psychological control will benefit from larger samples with multiple data waves to capture the likelihood that important interactive and cascading dynamics may be operating across parent, youth, and neighborhood influences on psychological control.

Fifth, including objective neighborhood data based on the youth’s city and zip code of residence represents a novel contribution to the literature on parental psychological control. However, future studies would benefit from a more granular methodological approach, such as virtual street audits using Google’s Street View tool to obtain block- and street-level observations of physical neighborhood disorder (Mooney et al., 2014). Likewise, because FBI and census data reflect a single time point, it was not possible to investigate potentially meaningful temporal dynamics as youth operate within their neighborhood contexts and parents respond to shifting neighborhood conditions. Here again, a more granular approach, such as geographical momentary assessment to track temporal shifts in neighborhood characteristics (Epstein et al., 2014) would be informative for future studies.

Finally, although this study demonstrated that youth externalizing problems are a significant predictor of maternal psychological control in early adolescence, evidence supporting significant relations of youth internalizing and social problems with parental psychological control (e.g., see Scharf and Goldner, 2018 for review) speaks to the need for future research studies that consider multiple facets of youth adjustment in predicting parental psychological control. Relatedly, as noted earlier, future studies should endeavor to identify shared and distinct predictors of both psychological *and* behavioral control practices, as well to clarify how each of these predictors influence parenting individually and interactively.

## Conclusion

Parental psychological control encompasses restricting and rejecting parenting practices that threaten positive youth development by thwarting autonomy and disrupting identity processes. Prior research has been limited by the use of cross-sectional research designs and single informant reports. The current multi-informant, multi-method longitudinal investigation found that youth and maternal perceptions of psychological control may have distinct precursors, such that maternal problems and youth externalizing problems predicted changes in maternal reports of psychological control across adolescence, whereas neighborhood risks predicted changes in youth reports of maternal psychological control. Exploratory findings by gender suggested that maternal problems predicted increased maternal reports of psychological control for boys only, while neighborhood risks predicted decreased maternal reports of psychological control toward girls, but increased maternal reports of psychological control toward boys. Adding to past research highlighting how, when, and for whom parental psychological control is detrimental for youth development (see Scharf and Goldner, 2018), the obtained findings support Belsky’s (1984) *process model of parenting*, which argues that multiple factors converge to influence parenting practices, while illuminating the potential for differential relations across informant and gender groups. Together, the current findings suggest that prevention and intervention applications to modify maladaptive parental behaviors, such as parental psychological control, may benefit from concurrent efforts to address parent and youth psychopathology, as well as community-based efforts to enhance neighborhood safety and resources.
